# GPU acceleration of a model-based iterative method for Digital Breast Tomosynthesis

**DOI:** 10.1038/s41598-019-56920-y

**Published:** 2020-01-08

**Authors:** R. Cavicchioli, J. Cheng Hu, E. Loli Piccolomini, E. Morotti, L. Zanni

**Affiliations:** 1 University of Modena and Reggio Emilia, Department of Physics, Informatics and Mathematics, Modena, 41125 Italy; 2 University of Bologna, Department of Computer Science and Engeneering, Bologna, 40126 Italy

**Keywords:** Medical imaging, Computational science, Computer science, Software

## Abstract

Digital Breast Tomosynthesis (DBT) is a modern 3D Computed Tomography X-ray technique for the early detection of breast tumors, which is receiving growing interest in the medical and scientific community. Since DBT performs incomplete sampling of data, the image reconstruction approaches based on iterative methods are preferable to the classical analytic techniques, such as the Filtered Back Projection algorithm, providing fewer artifacts. In this work, we consider a Model-Based Iterative Reconstruction (MBIR) method well suited to describe the DBT data acquisition process and to include prior information on the reconstructed image. We propose a gradient-based solver named Scaled Gradient Projection (SGP) for the solution of the constrained optimization problem arising in the considered MBIR method. Even if the SGP algorithm exhibits fast convergence, the time required on a serial computer for the reconstruction of a real DBT data set is too long for the clinical needs. In this paper we propose a parallel SGP version designed to perform the most expensive computations of each iteration on Graphics Processing Unit (GPU). We apply the proposed parallel approach on three different GPU boards, with computational performance comparable with that of the boards usually installed in commercial DBT systems. The numerical results show that the proposed GPU-based MBIR method provides accurate reconstructions in a time suitable for clinical trials.

## Introduction

Digital Breast Tomosynthesis (DBT) is a quite recent 3D Computed Tomography (CT) technique providing a volumetric breast reconstruction as a stack of 2D images, each representing a cross-sectional slice of the breast itself^1^. When compared with traditional 2D mammography, DBT has the advantage of separating the breast anatomical tissues, which can be overlapped in a mammography, and this generally reduces false negative diagnosis. At the same time, since the DBT source emits X-rays only from a small number of angles in an arc trajectory, DBT provides a low radiation dose comparable to the radiation dose used in a standard mammography. For this reason, DBT is appealing to the medical and scientific community as a breast screening routine^2,3^.

Until a few years ago, the introduction of DBT in a clinical setting has been slowed down by the absence of efficient algorithms for image reconstruction from a limited number of projections. It is well known that the traditional Filtered Back Projection (FBP)^4^ analytic algorithm amplifies noise and artifacts in the case of low-sampled data^3^. Iterative algorithms are known to perform better than FBP in the case of tomosynthesis data, but they require computational times incompatible with a clinical use^3,5^, since in clinical DBT examinations the breast reconstruction must be provided in 40 to 60 seconds. Nevertheless, the recent advent of low cost parallel architectures, such as Graphics Processing Units (GPUs), has provided the chance for a remarkable reduction of the image reconstruction time, making iterative methods a realistic alternative to analytic algorithms.

Among the different iterative approaches in X-ray CT (see^5^ for a detailed classification of iterative methods in CT), the so called Model-Based Iterative Reconstruction (MBIR) methods are now getting growing attention. In general, they try to model the acquisition process as accurately as possible, since they take into account system geometry, physical interactions of photons in the projections and prior information about the acquired volume. This approach produces better results than the traditional FBP in terms of quality of the reconstructed image and artifacts reduction, especially for low-dose or limited data X-ray CT. The MBIR methods can be described in a unifying framework as constrained or unconstrained minimization problems^5–7^, involving a fit-to-data function and a regularizing function, acting as a prior on the solution. A widely used regularization term, proposed in sparse tomography by^8^ and then used by many authors^9–14^, is the Total Variation (TV) function, whose edge enhancing properties are very effective on medical images. Herein, we consider the general constrained minimization formulation:

1 $${\rm{\arg }}\,\mathop{{\rm{\min }}}\limits_{x\ge 0}\,\frac{1}{2}LS(x)+\lambda TV(x)$$

where *LS*(*x*) is the least squares fit-to-data function, *TV*(*x*) denotes the Total Variation regularizer, *λ* > 0 is the regularization parameter and the request of non-negativity on the solution is due to physical constraints.

In the CT framework, very popular algorithms for solving (1) are based on alternate minimization of the two terms of the objective function of (1). They process few views at a time with Ordered Subset strategies for the least squares term in (1) and use a fixed step size gradient descent approach on the TV function^6,8,11,12,15^. However, the two steps strategy may have a rather slow convergence, while an algorithm that computes the new approximate solution in a single descent step, through the computation of the gradient of the whole objective function, generally has a faster convergence^16^. In sparse tomography, these gradient-based minimization methods have been used in^7,9,17^. In this work, we propose to solve (1) by an accelerated gradient scheme belonging to the class of Scaled Gradient Projection (SGP) methods^18,19^. The SGP methods have been recently applied in low-sampled X-rays cone beam CT (CBCT) image reconstruction, with very good results in terms of image accuracy^13,14,20^. In particular, in^14^ the authors proposed a SGP method for X-rays CT image reconstruction and applied it to a phantom simulation using a geometry different from DBT limited angles. Since SGP showed a very fast convergence in the first iterations, we choose a similar approach also for our DBT application.

Even if the SGP algorithm is expected to produce accurate reconstructions in few iterations, the high computational cost of each iteration still prevents the completion of the reconstruction process in a suitable time on a serial architecture. Exploiting the computational power of modern GPU boards, we aim to perform the expensive operations of each SGP iteration in a time consistent with the practical constraints imposed by the DBT application. Similar parallel approaches have been investigated in the case of 3D X-ray CT image reconstruction^9–11,15,21^, but none of these schemes is based on gradient methods accelerated by scaling strategies nor is optimized for the particular case of DBT data. To achieve our goal, we design a parallel SGP version in which the most time consuming task involved in each iteration, represented by the computation of the gradient of the objective function in (1), is distributed according to the hardware features of commonly available low cost GPU boards. The proposed implementation is evaluated in terms of time and image quality by reconstructing images from DBT simulated and real projections on three different GPU boards. The experiments show that the parallel SGP implementation performs a number of iterations suitable for reconstructing images with enhanced quality in about one minute (as usually required in clinical trials).

## Results

### Digital breast tomosynthesis imaging

Following the ongoing technological development, the medical imaging community is investing in innovation, looking for healthier and more reliable tools. To set an example, in traditional 2D mammography, cancerous masses are often camouflaged by the superposition of dense breast tissue on the final image, hence 3D breast reconstructions are getting increasing interest. On the other hand, the radiation risk from X-ray based medical imaging is a matter of fact, especially in classical 3D CT where hundreds of X-ray scans are performed from different points over a circular trajectory around the patient, in order to get a complete data set producing an accurate reconstructed image. As a consequence, classical 3D CT is not suitable for screening tests. The so-called tomosynthesis (a quite recent tomographic routine) tries to overcome this issue: it is characterized by the acquisition of a small number of projections captured from a limited angular range around the patient thus resulting in a faster and safer examination than traditional 3D CBCT^1^. As a matter of fact, DBT has been included in the diagnostic and screening settings in adjunct to digital mammography in some European countries, such as Italy^22,23^.

**Figure 1:**
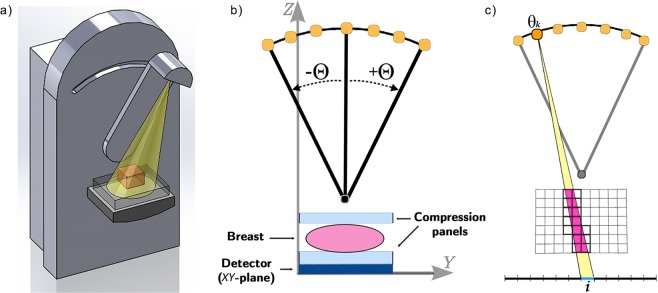
(**a**) Draft of the DBT system. (**b**) Picture of the DBT geometry mashed on the *YZ* plane. The breast is constricted by compression paddles which are parallel to the breast supporting table and the detector plane (*XY* plane). (**c**) A schematic draw of the projection process of the volume onto a single detector unit from a view in a mashed 2D representation on the *YZ* plane. Scanning from the $k-th$ position of the X-ray source, the yellow cone of X-rays represents the projecting rays on the $i-th$ pixel area (in blue). It intersects the object in the magenta volume, hence all the voxels contributing to this projection are highlighted by bold edges. The contribution of each voxel in the projection is proportional to the magenta portion inside the voxel itself.

Figure 1(a) illustrates a draft of the tomosynthesis device for the specific case of the breast imaging: the X-ray source emits a cone beam at each angle and its trajectory is restricted from the classical circular one to a C-shape path. In Fig. 1(b) we report a more technical draw of the DBT geometry mashed on the *YZ*-plane in order to better visualize the device setting, and in Fig. 1(c) we show the X-ray cone beam projection for one recording unit on the detector. Due to the lack of data, the resolution of the reconstructed images along the in-depth *Z*-direction is coarser than in the *XY*-plane. Moreover, the incomplete data sampling leads to well-studied artifacts. In particular, DBT images present two different types of artifacts: in-plane artifacts, consisting in a dark path in the direction of the source motion around the dense objects, and in-depth artifacts, appearing as *N*_*θ*_ shadows in the slices preceding and following the object along the *Z*-direction^24^.

More specifically, the quality of the reconstructed images depends on the number *N*_*θ*_ of acquired projections, typically from 10 to 25, and on the angular range, usually from 15 to 50 degrees.

### Materials

We have tested the proposed SGP method on two real data sets, fitting the European DBT Protocol^22^, obtained by the digital system Giotto Class of the IMS Giotto Spa company and on a synthetic data set obtained by simulating the projections of a digital phantom with the Giotto geometry. In Giotto apparatus, the reference system is set as in Fig. 1(b). In the central position, the X-ray source is approximately 69 cm above the detector and it shoots *N*_*θ*_ = 11 projections with a low dose protocol, in a very limited angular range from −15 to 15 degrees. The flat detector has 3580 × 2812 square recording units with edges equal to 0.085 mm and it is large enough to contain the widest compressed breast projections. As usual habit, in order to speed up the reconstruction phase, in a preprocessing step the projections are cut to eliminate those portions of the data which do not contain useful information for the breast reconstruction. If the resulting crop consists of *n*_*x*_ × *n*_*y*_ pixels for each projection, then the available data set has size *N*_*d*_ = *n*_*x*_ × *n*_*y*_ × *N*_*θ*_. The system uses a polychromatic ray with energies in a narrow range around 20 keV to avoid the photon scattering. As often happens in CT reconstruction algorithms, we approximate the polychromatic ray with a monochromatic one.

The first data set (D1) considered has size 3000 × 1500 × 11 and is obtained by scanning the *Tomophan Phantom TSP004*, by The Phanto Laboratory^25^. The phantom contains aluminum Test Objects (TOs) embedded into a clear urethane material and it has a semi-circular shape with a thickness of 42 mm. Due to its structure, this Quality Control (QC) accreditation phantom is used for different tests (see the phantom data sheet^25^). Among them, we concentrate on evaluating the homogeneity of the in-plane aluminum objects, the contrast between the luminescent aluminum objects and the dark background, and the measure of the noise in the background. In particular, in the reconstructed image we will focus on three 0.500 mm aluminum beads spaced 10 mm along the *Z*-direction and hence positioned on three different layers.

The second data set tested (D2) has size 3000 × 1500 × 11 and it is obtained by scanning the *BR3D Breast Imaging Phantom*^26^ (model 020), produced by the CIRS company, which is widely used to assess the detectability of lesions of different sizes. The objects of interests, constituted by microcalcifications, fibers and masses, are within a heterogeneous background composed by epoxy resin to simulate pure adipose and pure glandular tissues, mixed together to mimic a real breast. In particular, we will analyze the reconstruction of a cluster of CaCO3 small beads, simulating microcalcifications of 0.290 mm in diameter.

Finally, we have tested the SGP algorithm on a simulated data set (D3), obtained from a digital phantom we designed. The D3 data set has 3200 × 1100 × 11 elements, computed as projections of the digital phantom under the geometric setting of the Giotto Class device onto a detector with 0.100 mm element pitch. We added Gaussian noise with SNR = 50 to the computed projections (where the SNR value is computed as the logarithm of the ratio between the noisy projections and the noise, multiplied by a factor of 20), to better simulate a real X-ray acquisition. Our phantom reproduces the main features of the *BR3D Breast Imaging Phantom*: it contains several test objects reproducing microcalcifications, fibers and masses, immersed into a uniform adipose-like background. The attenuation coefficients of the test objects and background are taken from a reconstruction of the *BR3D Breast Imaging Phantom* performed on a commercial system. The analysis on the reconstructed images carried out in the next subsection will concentrate on the detection of a cluster containing 0.300 mm diameter beads, simulating microcalcifications of medium size.

The data sets analysed during the current study are available at the website: https://cloud.hipert.unimore.it/s/ptqjtMdwXA7sc5N.

### Reconstructions

In this paragraph we evaluate the quality of the reconstructions obtained with the proposed SGP method on the data sets presented above. We are interested in monitoring how the algorithm performs for an increasing number of iterations, hence we focus on the reconstructions achieved in 4, 12 and 30 iterations.

The volume to be reconstructed is discretized into *N*_*v*_ = *N*_*x*_ × *N*_*y*_ × *N*_*z*_ volumetric elements (voxels), where *N*_*x*_, *N*_*y*_, *N*_*z*_ are the number of elements along the three Cartesian directions. We observe that in DBT imaging *N*_*v*_ > *N*_*d*_. The reconstructions of real phantoms have in-plane voxel size of 0.090 mm, whereas the in-depth one is 1.000 mm. In all the tests, the regularization parameter *λ* has been tuned by a trial and error procedure. The images presented in this section show slices of the reconstructed volume parallel to the *XY*-plane. We remind that the source moves along the C-shaped path on the *Y* direction. For each phantom, the intensities of the reconstructions are shown in the same gray scale (as arbitrary unit).

#### Tomophan phantom results

**Figure 2:**
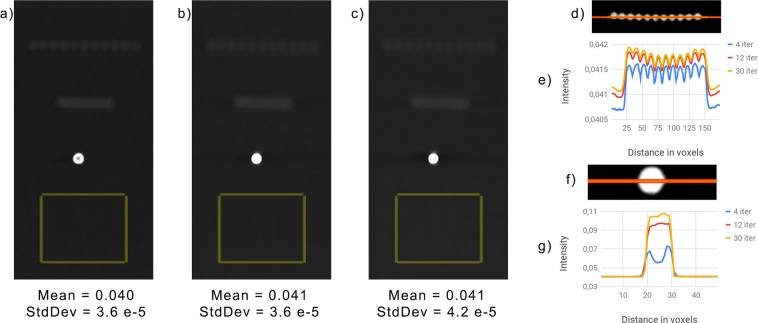
(**a**–**c**) Crops of the reconstructions from data set D1, provided in 4, 12 and 30 iterations by the SGP algorithm, respectively. They are represented in the same gray scale. The yellow rectangle is the uniform area for the computation of the mean and standard deviation (StdDev) of the background. (**d**) The in-plane profile of interest on the metal artifacts. (**e**) Comparison between the profiles of interest in (**d**) at different iterations. (**f**) The in-plane profile of interest on the reconstructed aluminum bead. (**g**) Comparison between the profiles of interest in (**f**) at different iterations. In (**e**,**g**) the blue, red and yellow line corresponds to the solution at 4, 12 and 30 iterations, respectively.

We report in Fig. 2 some results obtained on the D1 data set. Figure 2(a–c) show the reconstructions after 4, 12, 30 iterations inside a region of 160 × 320 pixels (corresponding to 14.4 mm × 28.8 mm) on the layer 19, where the lower metallic sphere is on focus. The yellow rectangle in figures (a–c) defines the region where we computed the mean and the standard deviation (StdDev) values reported under each corresponding figure in order to analyze the background uniformity and the noise. Above the bead, we can also see the expected metallic artifacts produced by the two specks located on layers 39 and 29. The artifacts corresponding to the highest speck are in the form of 11 (i.e., the number of projections views) small circles, while the artifacts produced by the central sphere are in the form of a light strip. The intensity of these artifacts can be analysed on the profile identified by the orange line of Fig. 2(d); the reconstructions of the profile at different iterations are compared in Fig. 2(e).

As concerns the in focus bead, we study the orange profile in Fig. 2(f) compared among the three considered reconstructions in Fig. 2(g). The blue line shows that after 4 iterations the recovered values inside the sphere are quite low and non-uniform, whereas the bead detection improves getting higher values of about 0.092 and 0.115 in the reconstructions at 12 and 30 iterations, respectively.

#### BR3D breast phantom results

**Figure 3:**
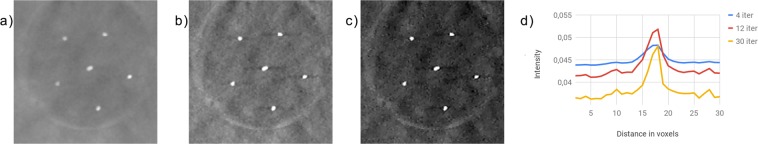
(**a**–**c**) Crops of the reconstructions from data set D2, provided by the SGP algorithm in 4, 12 and 30 iterations, respectively. They are represented in the same gray scale. (**d**) Comparison between the in-plane profile of interest along the *Y* axis of the central bead at different iterations. The blue, red and yellow line corresponds to the solution after 4, 12 and 30 iterations, respectively.

Now we analyse the results obtained from the data set D2, characterized by breast-like background and objects of interest. They are perfectly on focus on the selected layer. In Fig. 3(a–c) we report the reconstruction crops corresponding to a 145 × 145 pixel region (13.05 mm × 13.05 mm) containing a cluster of microcalcifications. In Fig. 3(d) we plot the corresponding in-plane profiles on the central speck along the *Y*-direction.

#### Digital phantom results

**Figure 4:**
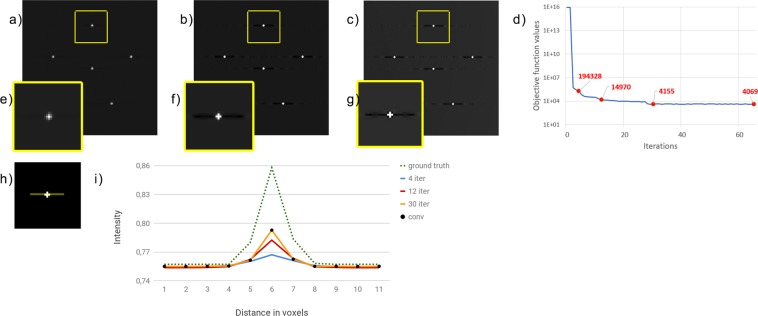
(**a**–**c**) Crops of the reconstructions from the simulated data set D3, provided by the SGP algorithm in 4, 12 and 30 iterations, respectively. They are represented in the same gray scale. (**d**) Plot of the objective function (1) versus the number of iterations. The red labeled dots report the values at 4, 12, 30 iterations and at convergence. (**e**–**g**) Magnified views of the object inside by the yellow box for each reconstruction. (**h**) The profile of interest along the *Y*-direction. (**i**) Comparison between the in-plane profiles at different iterations and the ground truth. The green dotted profile shows the ground truth target, the blue, red and yellow line corresponds to the reconstructed profile at 4, 12 and 30 iterations; the black dots show the profile obtained when the convergence criterion is met.

In order to better analyse the accuracy of the MBIR approach in the challenging task of a microcalcification detection, we present here the results obtained on the simulated D3 data set. The digital phantom to be reconstructed from the D3 data set has size 3000 × 1000 × 50 with voxel size of 0.100 mm along the *X* and *Y* axes and 1.000 mm along the *Z* direction. Figure 4(a–c) show the results obtained from D3 inside the 173 × 173 pixel ROI (corresponding to 17.3 mm × 17.3 mm) of the central layer of the synthetic phantom, containing small microcalcifications. We have zoomed over one microcalcification in Fig. 4(e–g) where we can appreciate how the 3-voxel wide object is already detected after only 4 iterations. Fig. 4(d) shows the behaviour of the objective function minimized in (1) with respect to the iteration number. In Fig. 4(i) we plotted the profile of the yellow line of Fig. 4(h) reconstructed after 4, 12 and 30 iterations and, with black dots, the solution obtained when the convergence criterion is met (i.e., after 64 iterations). The green dotted line corresponds to the exact profile.

### Parallel executions

The reconstructing software has been implemented in C code on a commercially available high end computer, equipped with Intel i7 7700K CPU at 4.2 GHz, 32 GB of RAM and 1 TB of Solid State Disk (SSD), and its parallel implementation has been performed on NVIDIA GPUs by means of the CUDA SDK^27^. The program has been tested on different GPUs, with different memories, number of CUDA cores and, obviously, price point.

In details, we have considered the following GPU boards:

- GTX 1060: 6 GB of RAM, 1280 CUDA cores, launch price 250$;
- GTX 1080: 8 GB of RAM, 2560 CUDA cores, launch price 700$;
- Titan V: 12 GB of RAM, 5120 CUDA cores, launch price 3000$.

We tested two different parallel implementations on the Titan V board: the first one (denoted as Titan V_1) has the same approach considered for the GTX 1060 and GTX 1080 boards, where the data cannot be fully stored in RAM and many data transfers between the CPU and the GPU are required during each SGP iteration, while the second (denoted as Titan V_2) exploits the larger GPU RAM to store all the needed data. For more details on the parallel implementations, please refer to the corresponding section.

The results shown in this paragraph are obtained on the D3 synthetic data set, whose volume is made of *N*_*v*_ = 1.5·10^8^ voxels. We have identified four main tasks in the SGP algorithm: the Forward and Backward projections, the TV evaluation and all the remaining operations (*Other*), mainly consisting of scalar products and vector sums. We will analyse the parallel algorithm performance through these four tasks.

**Figure 5:**
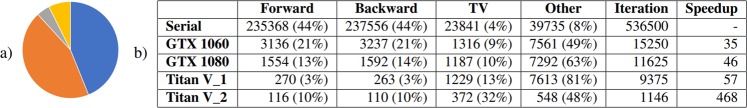
Computational time of a single SGP iteration (**a**) Pie chart of time spent for the four considered kernels: Forward projection in blue, Backward projection in orange, TV evaluation in gray and all remaining computations in yellow. (**b**) The table reports in each row the computational time of the four considered kernels and, in brackets, their percentage with respect to the whole iteration time (column 6); the resulting speedup is reported in column 7. All the times are in milliseconds.

By profiling a serial execution of the program, we can draw the pie chart in Fig. 5(a) of the computational time for the different tasks composing a single SGP iteration (for a comprehensive description of the algorithm, please see the Methods sections): 88% of the time is spent for the Forward and Backward projections, 4% for the evaluation of the TV function and of its gradient and 8% for all remaining SGP operations.

Figure 5(b) shows the execution times in milliseconds per iteration for each of the 4 computational kernels; the last column reports the global speedup. The time required for a Forward or Backward projection decreases from about 4 minutes in the serial execution to 1–3 seconds on the GTX boards and to less than 300 milliseconds for the Titan V_1 case. The parallelization of the TV function reduces the time from 23 seconds to 1 second, while the execution time for *Other* decreases from about 40 to 7 seconds: these times are almost constant on all the three boards since they are mainly due to data communications between CPU and GPU, which is independent from the considered GPU board. Different performances are provided by the Titan V_2 case which achieves a speedup of almost 470. We remark that the reported times correspond to an SGP iteration with only one execution of the inner backtracking loop; each further backtracking step requires to evaluate only the TV component of the objective function, but it does not involve Forward or Backward projections.

**Figure 6:**
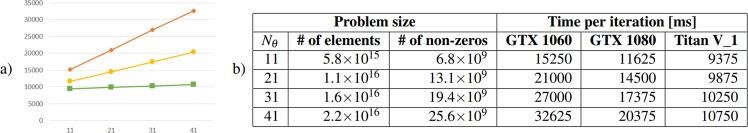
Results for an increasing number of projection angles. (**a**) Computational time as a function of the number of projection angles for the different boards. GTX 1060 is represented with an orange line (diamonds), GTX 1080 with a yellow line (dots) and TitanV_1 with a green line (squares). (**b**) Table containing in each row the information corresponding to a data set with ${N}_{\theta }$ projection angles: in columns 2 and 3 number of elements and of nonzeros entries of the projection matrix *A*; in columns 4, 5 and 6 computational times in milliseconds.

Figure 6 shows the time per iteration on the three considered GPUs for data sets simulated by projecting the digital phantom used in D3 with a varying number of angles *N*_*θ*_ ∈ {11, 21, 31, 41}.

## Discussion

Looking at Fig. 2 and recalling the characterizations of the Tomophan Phantom, we can make the following remarks. It is evident from Fig. 2(a–c) that for increasing iterations the contrast between the lower aluminum sphere and the background enhances. Moreover, when the algorithm approaches the solution of (1), the sphere gets more and more homogeneous inside, as can be viewed by the plots in Fig. 2(g). The mean and standard deviation reported under Fig. 2(a–c) confirm that the noise keeps very low along the iterations. This proves that the TV regularization term efficiently smooths the noise and that a suitable value for the regularization parameter has been chosen. There are visible artifacts in the upper part of the images which are due to the small number of projection angles. However, from the plots in Fig. 2(e) we observe that their amplitude is very small and their contrast from the background decreases with ongoing iterations. We also notice that all the artifacts are spread along the *Y* axis direction according to the X-ray source motion. From all the above considerations, we can conclude that increasing the iterations from 4 through 30 improves the quality of the reconstructed image for this test phantom.

The images in Fig. 3(a–c) show how the small beads of the microcalcification cluster are detected by the solver after very few iterations, but with low contrast from the background. After 30 iterations, the contrast is far enhanced. Figure 3(d) provides a double piece of information on solution changes during the iterations: it confirms that the contrast improves with the increasing number of iterations and that the microcalcification width gets closer to the expected one of 290 micrometers (corresponding to about 3 voxels wide).

By analysing the results of the D3 data set we compare the reconstructed images with a ground truth. The regularizing effect of the TV prior is visible in Fig. 4(e–g), where the object spread is progressively reduced in favour of a higher contrast with the background. Figure 4(i) confirms that values obtained for the microcalcification get closer to the ground truth by increasing the number of iterations, whereas the exact background is recreated from the first iterations. Since the black dots, representing the solution when the convergence criterion is met, overlap the 30 iteration profile, we can conclude that the reconstruction after 30 iterations is the best possible to obtain. We can also point out that the plot in Fig. 4(d) shows a remarkable decrease of the objective function in the first iterations. As expected, there is a discrepancy between the computed solution and the exact one which is mainly due to the sub-sampling of data in the DBT acquisition process.

From the performed tests we can conclude that the proposed model-based method, considering the L2-TV mathematical model and using the SGP optimization algorithm, provides accurate DBT image reconstructions in few tens of iterations. Concerning the comparison with other iterative optimization algorithms, the SGP algorithm exploits a scaling acceleration strategy for improving the performance of the standard Gradient Projection Barzilai-Borwein (GPBB) method^14^ and the GPBB method has been proved to outperform widely used alternating minimization methods in the work by Park *et al*.^9^.

As we stated in the Introduction, the drawback of model-based methods is the long computational time required. From the results presented in Fig. 5 we can notice that the computational times are dramatically reduced in the parallel algorithm execution, with speedups ranging from 35× to 57× thanks to increasing computational power for GTX1060, GTX1080 and Titan V boards. The execution of the Titan V_2 version further breaks down the time, getting a speedup of almost 500, due to the particular implementation that avoids most of the data transfer between the CPU and the GPU memory. Figure 6 proves that our parallel implementation linearly scales with the size of the problem, providing different slopes for the different boards. In particular the Titan V_1 has an incremental slope of 50 with respect to the GTX 1060 which is about 600, showing an enhanced scalability. We can state that the proposed parallel implementation drastically cuts down the computational time, making the execution times for the reconstruction of real volumes compatible with clinical requirements. This is possible thanks to the ability of the SGP algorithm to provide suitable reconstructions in a few tens of iterations, which can be performed in less than one minute by the most recent boards, like the Titan V. Taking into account that the market of GPUs rapidly evolves towards more and more powerful and less costly boards, the proposed MBIR approach represents a useful tool for achieving the goal of getting large volumetric images of superior quality at affordable costs in the near future.

## Methods

### Scaled gradient projection method

In this paragraph we briefly describe the serial version of the SGP algorithm which has been implemented to solve the minimization problem (1) for DBT reconstruction. The SGP method is a first-order descent method for the solution of a general minimization problem of the form: ${\rm{\arg }}\,{{\rm{\min }}}_{x\ge 0}f(x)$, where $x\in {{\mathbb{R}}}^{n}$ and $f:{{\mathbb{R}}}^{n}\to {\mathbb{R}}$ is a convex differentiable function. In order to accelerate the classical Gradient Projection method, SGP introduces at each iteration *k* a scaling procedure^18^, by multiplying the descent direction $-\nabla f({x}^{(k)})$ by a diagonal matrix *D*_*k*_ with entries in the interval $[\frac{1}{{\rho }_{k}},{\rho }_{k}],{\rho }_{k} > 1$, (we call ${ {\cal{D}} }_{{\rho }_{k}}$ the set of these matrices). Moreover, SGP exploits Barzilai-Borwein^28^ type rules for the choice of the steplength to ensure a fast decrease of the objective function.

In details, after initializing ${x}^{(0)}\ge 0,\gamma ,\sigma \in (0,1),0 < {\alpha }_{{\min }}\le {\alpha }_{{\max }},{\alpha }_{0}\in [{\alpha }_{{\min }},{\alpha }_{{\max }}]$, ${\rho }_{0} > 0$, ${D}_{0}\in { {\cal{D}} }_{{\rho }_{0}}$, the following steps are repeated for *k* = 0, 1… until a stopping criterion is satisfied.

1. Compute the scaled descent projected direction *d*^(*k*)^ as ${d}^{(k)}={P}_{+}({x}^{(k)}-{\alpha }_{k}{D}_{k}\nabla f({x}^{(k)}))-{x}^{(k)}$, where *P*+(*z*) is the Euclidean projection of the vector $z\in {{\mathbb{R}}}^{n}$ onto the non negative orthant.
2. Perform a backtracking on the computed direction *d*^(*k*)^ starting with *η* = 1:
  $$\begin{array}{c}while\,f({x}^{(k)}+\eta {d}^{(k)}) > f({x}^{(k)})+\sigma \eta \nabla f{({x}^{(k)})}^{T}{d}^{(k)}\\ \eta =\gamma \,\eta ;\end{array}$$
3. Compute the new iterate: ${x}^{(k+1)}={x}^{(k)}+\eta {d}^{(k)}$.
4. Update ${\rho }_{k+1}=\sqrt{1+{10}^{15}/{(k+1)}^{2.1}}$ and the diagonal scaling matrix ${D}_{k+1}\in { {\cal{D}} }_{{\rho }_{k+1}}$.
5. Update the steplength ${\alpha }_{k+1}\in [{\alpha }_{min},{\alpha }_{max}]$.

The update of the scaling matrix *D*_*k*+1_ is performed through a splitting of the gradient of the objective function in its positive and negative parts as in^14^ and the definition of *ρ*_*k*+1_ is aimed at avoiding restrictive bounds on the diagonal entries of *D*_*k*+1_ in the initial phase of the iterative process and satisfying the SGP convergence conditions^19^ by asymptotically forcing *D*_*k*+1_ towards the identity matrix. The update of the steplength *α*_*k*+1_ is obtained by using an alternate Barzilai-Borwein strategy; in particular, we use adaptive alternations of the two classical Barzilai-Borwein rules proposed in^29^ and applied also in^14^.

The algorithm is stopped when:

2 $$|f({x}^{(k)})-f({x}^{(k-1)})| < {10}^{-6}|f({x}^{(k)})|\,{\rm{o}}{\rm{r}}\,k > {maxiter}$$

where *maxiter* is the maximum number of iterations allowed. For more implementation details and for the convergence properties of SGP refer to^18,19^. Even if the theoretical convergence rate ${\mathscr{O}}(1/k)$ on the objective function values is lower than the rate ${\mathscr{O}}(1/{k}^{2})$ of some optimal first-order methods^17,30^, the practical performance of SGP method is very well comparable with the convergence rate of the optimal algorithms^19^.

### Discrete mathematical model

The MBIR methods for CT image reconstruction are built on the discretization of the Lambert-Beer law, relating the acquired and emitted intensities of X-rays^31^ in the tomographic process. The resulting model is a linear system of the form *Ax* = *b* where *b* collects all the ${N}_{d}={N}_{\theta }\times {n}_{x}\times {n}_{y}$ tomographic noisy projections, acquired during ${N}_{\theta }$ X-ray scans as ${n}_{x}\times {n}_{y}$ projection images, lexicographically reordered into a vector shape; the unknown *x* is a *N*_*v*_-dimensional vector resulting from the 3D discretization of the volume. The operator projecting a volume $x$ onto the detector according to the geometry of the CT device is called Forward Projection (FwP). This is mathematically modeled by computing an ${N}_{d}\times {N}_{v}$ coefficient matrix *A*, where each element represents the geometrical contribution of one voxel to one pixel of the detector for each projection angle, and multiplying the matrix by a *N*_*v*_-dimensional vector of the object space. The operator acting from the projection space to the object space is called Backward Projection (BwP) and it is implemented via multiplication of *A*^*T*^ by a *N*_*d*_-dimensional vector of the projection space.

In the particular case of DBT, due to the sub-sampling of the limited angle geometry, ${N}_{d} < {N}_{v}$, hence the linear system $Ax=b$ is under-determined and admits infinite solutions. In order to force the uniqueness of the solution and enhance the quality of the reconstruction, the problem is formulated as a minimization task as in (1), where the objective function is defined as:

3 $$f(x)=\frac{1}{2}LS(x)+\lambda TV(x)=\frac{1}{2}\parallel Ax-b{\parallel }_{2}^{2}+\lambda \mathop{\sum }\limits_{j=1}^{{N}_{v}}\sqrt{\parallel \nabla {x}_{j}{\parallel }_{2}^{2}+{\beta }^{2}}.$$

Here, $\parallel \nabla {x}_{j}{\parallel }_{2}$ is computed with respect to the three Cartesian directions with forward differences, while *β* > 0 is a small constant introduced to overcome the non-differentiability of the discrete TV function whenever $\parallel \nabla {x}_{j}{\parallel }_{2}$ is zero for some *j*.

The function *f*(*x*) defined in (3) represents the objective function of the constrained minimization problem that we solve with the SGP method, whose gradient is: $\nabla f(x)={A}^{T}Ax-{A}^{T}b+\lambda \nabla TV(x)$. We remark that in, the serial execution, the main computational cost at each SGP iteration is given by one FwP and one BwP involving the matrix-vector product by *A* and ${A}^{T}$ in the gradient formula (as shown in Fig. 5(a)). Moreover, the matrix is too large to be stored in central memory and it must be re-evaluated at each matrix-vector operation (further details in “Parallel implementation”). The computation of the matrix *A* plays a key role in the accuracy of the CT modeling. Among the several approaches available in literature to define the tomographic matrix, we considered the *distance-driven* (DD) one, proposed for the 3D geometry in 2004^32^: it fits very well the CBCT geometry and is suitable to be split among many processors. The draft in Fig. 1(c) schematically represents the DD Forward Projection process for one pixel *i*, in a 2D scheme of the coronal view. In particular, fixed an angle ${\theta }_{k}$ and a pixel *i*, the corresponding row of *A* contains the weights representing the contribution of all the voxels to the projection onto the pixel *i*. To compute the weights, the DD algorithm first identifies which voxels are crossed by the X-rays reaching the $i-th$ pixel (the voxels with bold edges in Fig. 1(c)); then it quantifies the contribution of all and only the identified voxels as the volume of the intersection between each voxel and the X-ray beam. We observe that *A* is a very sparse matrix; in our tests, it always has a density factor of the order of 10^−7^. To compute the $\nabla TV(x)$ function in each voxel, we used forward differences involving the voxel values^14^.

### Parallel implementation

The proposed SGP method is suitable for parallel implementation on GPU as reported in^33,34^. We describe in this section the parallel implementation of the SGP method for DBT image reconstruction. We exploit the massive parallel architecture of the GPUs by distributing the work to hundreds of small cores. A high level of parallelism is achieved by exploiting hundreds of computing pipelines, usually grouped into processing clusters, which can work both in single and double precision. Within each of these processing clusters, one hardware scheduler dispatches small groups of threads to the pipelines. To manage the communications between the CPU and the GPU, the division of the work and the partition of thread in blocks, we use CUDA^27^, a well-known Application Programming Interface developed by NVIDIA.

**Figure 7:**
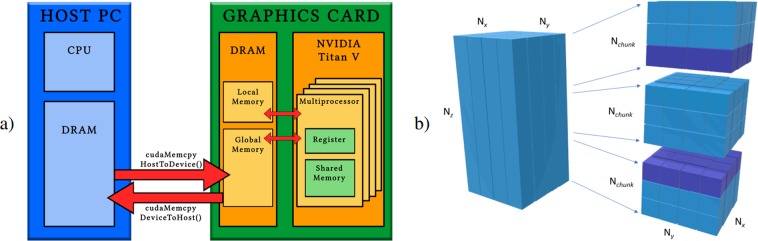
(**a**) Logical view of a system composed by host and accelerator. The data which are stored in the host memory (DRAM) must be transferred to the graphics card memory (Global Memory) to perform the computations and subsequently the results must be transferred back to be saved in DRAM. (**b**) Schematic draw of the partitioning into chunks of a volume. The data (on the left) are divided into ${N}_{z}/{N}_{chunk}$ chunks. Each chunk is sent and processed independently in the SGP parallel kernels. Only in the execution of the kernel computing the TV function and its gradient, the purple slices of adjacent blocks are added to each chunk for the computation of the finite differences on the border elements.

As already observed, the typical size of the data is so large that storing the whole matrix *A* in RAM is unfeasible. For example, in the data set D3 this would amount to (3000 × 1000 × 50) × (3200 × 1100 × 11) = 5.8·10^15^ elements (about 46 Petabytes in double precision data), which is much larger than the capacity of the most recent RAM. Furthermore, the number of non-zero elements (see Fig. 6(b)) makes the storage of *A* in a sparse matrix data structure inconvenient, since it would involve too large data transfer from the CPU memory to the GPU one, representing a crucial bottleneck for the GPU performance (see Fig. 7(a)). In D3 data set, a sparse matrix data structure representation requires at least 110 GB and the considered GPUs take about ten seconds for the memory transfer (almost an order of magnitude more than our approach). As a consequence, we organize the parallelization of the FwP and BwP operations by computing the non-zero entries of *A* every time we need them.

Fixed ${N}_{thread}$ as the number of threads per block, we created a grid of $\lceil {N}_{v}/{N}_{thread}\rceil$ blocks. Within this configuration, each thread *i* independently computes the $i-th$ row of the system matrix *A* according to the DD strategy.

In the FwP evaluation, each thread *i* computes the product between the $i-th$ line of *A* and the column vector and saves the result in the $i-th$ position of the resulting vector. Due to the independence of all these computations, we do not need any data access synchronization. Inside the BwP step we call exactly the same DD function to compute the matrix *A*, hence we perform the product involving ${A}^{T}$ in a vector manner. Each thread *i* still computes one row of *A* (i.e., column of ${A}^{T}$), it multiplies the contribution of each $j-th$ voxel to the $i-th$ element of input vector and atomically sums the result to the $j-th$ element of output vector.

For the parallelization of the TV and its gradient, we only need to operate on the values of the voxels. However, the data access pattern is not coalesced and this limits the speedup of its implementation. All remaining computations in the SGP steps (previously labelled by *Other*) are vector operations and they are mainly executed in parallel with CUBLAS library functions provided within the CUDA environment.

All the computations are performed in double precision arithmetic, since the results obtained with single precision numbers were not satisfying. As a result, we need more than 11 GB of GPU memory to process the D3 data set. Since not all the commercial GPUs are equipped with such a large amount of memory, each parallel task in the Fwp, BwP, TV and *Others* is separately executed on the GPU on portions of data (called *chunks*) and the final results are collected on the CPU memory. Hence, the vectors of size ${N}_{v}$ or ${N}_{d}$ involved in the parallel computations are divided into *chunks* of size ${{N}}_{x}\times {{N}}_{y}\times {{N}}_{chunk}$ and ${n}_{x}\times {n}_{y}\times {n}_{chunk}$, respectively, where both ${{N}}_{chunk}$ and ${n}_{chunk}$ are integers depending on the memory available on the device. An example is depicted in Fig. 7(b). Since the amount of data transferred between the CPU and the GPU is constant, the number of chunks does not have a significant impact on overall performance.
